# Three-dimensional imaging for thoracoscopic resection of complex lung anomalies

**DOI:** 10.1186/s40792-017-0383-2

**Published:** 2017-09-25

**Authors:** Yuhei Yokoyama, Masaaki Sato, Mitsugu Omasa, Hiroshi Date

**Affiliations:** Department of Thoracic Surgery, Kyoto University, 54 Shogoin-Kawahara-cho, Sakyo-ku, Kyoto, 606-8507 Japan

**Keywords:** Thoracic surgery, Congenital lung disease, Three-dimensional computed tomography

## Abstract

**Background:**

Building surgical strategies for complex lung anomalies such as congenital pulmonary venolobar syndrome is difficult because of their rarity and variance. Using three-dimensional computed tomography (3D–CT), we can determine strategies safely. We describe a 27-year-old man with multifocal pulmonary malformations who underwent video-assisted thoracoscopic surgery (VATS) using 3D–CT.

**Case presentation:**

A 27-year-old man presented with hemoptysis associated with complex pulmonary malformations, including a triple-arched vein connecting the superior and inferior pulmonary veins with partial drainage into the inferior vena cava, a systemic and numerous arterial supply to the right lower lobe from the abdominal aorta, abnormal lobulation, and a tracheal bronchus in the right lung. Preoperative simulation using 3D–CT helped to determine the optimal surgical strategy, allowing for successful and safe completion of semi-emergent middle and lower bilobectomy via minimally invasive VATS.

**Conclusions:**

Preoperative simulation using 3D–CT helped to determine the optimal surgical strategy, allowing for successful and safe completion of surgery even in a complex case.

## Background

Many reports have described safe surgical techniques for the treatment of congenital lung abnormalities such as congenital venolobar syndrome [1, 2]. However, the most suitable operative procedure may need to be determined by intraoperative findings because of the unusual anatomy in such patients.

We report on a 27-year-old man with multifocal pulmonary malformations who underwent video-assisted thoracoscopic surgery (VATS). Although we encountered this unusual condition for the first time, preoperative simulation using three-dimensional computed tomography (3D–CT) and virtual bronchoscopy helped to determine the optimal surgical strategy.

## Case presentation

**Fig. 1:**
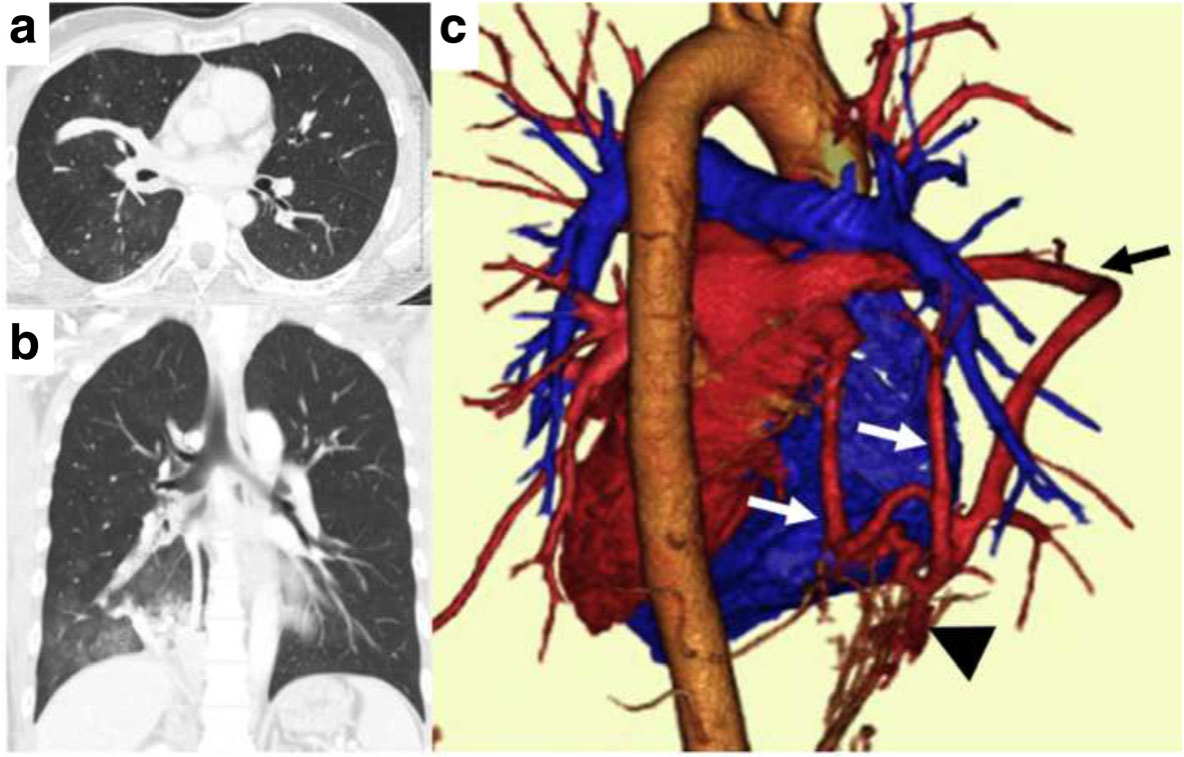
Multiple anatomical abnormalities demonstrated by CT images and their 3D reconstruction. **a** Axial-plane CT reveals the presence of an arched vein contiguous with the superior and inferior pulmonary veins. **b** Coronal-plane CT shows a tracheal bronchus. **c** Three-dimensional reconstructed image demonstrates anomalous venous drainage (*arrows*) and a systemic arterial supply from the abdominal aorta through the diaphragm (*arrowhead*). Note that there are three main anomalous venous arches penetrating the right lung almost regardless of the lobes and connecting to one another at the bottom of the lung. Venous drainage from the upper lobe is preserved and relatively normal. Numerous abnormal arteries are coursing from the systemic circulation at the diaphragm; there might be a direct connection between these arteries and the anomalous venous system

A 27-year-old man was referred to our hospital after presenting with hemoptysis. Plain radiography revealed right hilar anomalies. A chest CT scan demonstrated consolidation in the right lower lobe, and congenital lung and vascular anomalies, including bronchial malformations (tracheal bronchus), abnormal pulmonary venous drainage (triple-arched vein connecting the superior and inferior pulmonary veins with partial drainage into the inferior vena cava), and a systemic and numerous arterial supply to the right lower lobe from the abdominal aorta through the diaphragm (Fig. 1). In light of his clinical presentation, we elected to perform semi-emergent surgery.

**Fig. 2:**
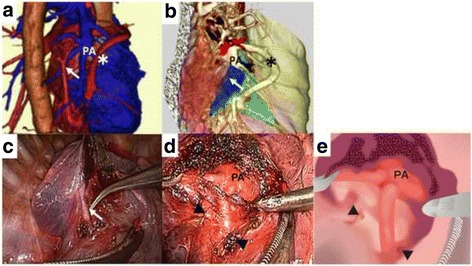
Preoperative 3D images and corresponding intraoperative findings. **a** Three-dimensional image shows an abnormal vein (*arrow*) running over a branch of the pulmonary artery. **b** An overlay of each lobe (shown in *different colors*) and a relatively well-developed fissure (marked *blue*), which is similar to that between the superior and basal segments of the lower lobe. The interlobar vascular structures seemed easiest to approach from this fissure (*arrow*). **c** Corresponding intraoperative view shows the exposed abnormal pulmonary vein (*arrow*; same vein as shown in **a**). **d** After transecting the abnormal vein shown in (**c**) (*arrowheads*), the interlobar pulmonary artery behind was approached according to the 3D images. **e** A traced illustration of (**d**). Asterisks in (**a**) and (**b**) indicate the same arch of an abnormal pulmonary vein

We quickly planned the operative procedure using 3D images and virtual bronchoscopy, constructed from enhanced thin-slice CT images using computer software (Aquarius iNtuition Client Viewer; TeraRecon, Inc., Tokyo, Japan), which clearly showed the unique vascular and bronchial anatomy of this patient. In particular, the abnormal pulmonary veins were running in disorder, penetrating multiple lobes. Although pneumonectomy appeared to be the first-choice operation, careful examination using 3D images indicated that the right upper lobe could be preserved safely (Fig. 1c). We decided not to preserve the middle lobe because of the risk of thrombus development in the abnormal vein and resultant systemic thromboembolism. Demarcation of the relatively well-developed interlobar fissure also was helpful to ensure a safe approach to the interlobar vascular structures, although the locations of the fissures also were abnormal (Fig. 2a, b).

Surgery was performed by complete VATS. Intraoperative findings included a hypoplastic right pulmonary artery, complex anomalous lobulation, and anomalous venous return with veins bridging the diaphragm. All of these intraoperative findings were consistent with the preoperative 3D images, allowing for safe completion of the right middle and lower lobectomies. The postoperative course was uneventful and the clinical course remained uneventful at the 2-year follow-up visit.

### Discussion

Congenital pulmonary venolobar syndrome is characterized by combined lung and vascular anomalies. In cases involving large abnormal draining veins, such as “meandering veins,” conventional open thoracotomy or median sternotomy often is required [1, 2]. Conversely, if possible, excessive operative procedures, such as pneumonectomy, should be avoided. Accurate preoperative evaluation is important to determine the most suitable surgical approach.

Several articles have reported the feasibility of 3D imaging in patients with congenital lung disease [3]. Most congenital lung malformations are diagnosed in adulthood, but can be treated effectively with minimally invasive surgical techniques [4]. From the viewpoint of minimally invasive surgery, preoperative evaluation is the most important factor in patients with congenital lung malformations. Preoperative pulmonary angiography and evaluation of the pulmonary-to-systemic flow ratio (Qp/Qs) may have use in revealing vascular abnormalities [5, 6]; however, this approach was considered to be unnecessary as long as the abnormal connection between the systemic and pulmonary circulation is identified and surgically excised.

Instead, as reported previously [7], we used 3D–CT and virtual bronchoscopy as preoperative evaluation to familiarize ourselves with the anatomy of our particular patient (Figs. 1c, 2). Moreover, by accurately accessing the vascular anatomy and lobulation of the lung, we preoperatively decided to preserve the upper lobe while considering the risk of systemic thrombosis, which might have occurred if we had preserved the abnormal drainage veins, particularly in the middle lobe in this case. Additionally, this meticulous preoperative assessment allowed us to safely approach the interlobar pulmonary tissue through the abnormal interlobar fissure (Fig. 2c, d). As a result, we were able to safely and confidently accomplish complete VATS as if we had been very familiar with this unusual anatomy. Such an approach utilizing 3D–CT would be useful not only in open surgery as well as in VATS.

## Conclusions

In conclusion, preoperative evaluation by 3D–CT and virtual bronchoscopy enabled identification of the most suitable minimally invasive surgical approach despite the presence of a complex multifocal malformation in the setting of a semi-emergent procedure.

## Abbreviations

3D–CT: Three-dimensional computed tomography
CT: Computed tomography
VATS: Video-assisted-thoracoscopic-surgery

## References

[1] Nuebel J, Januszewska K, Loeff M, Theisen D, Malec E, Dalla-Pozza R (2010). Unique technique of surgery in an unusual variety of scimitar syndrome: a case report. J Cardiothorac Surg.

[2] Lugones I, Garcia R (2014). A new surgical approach to scimitar syndrome. Ann Thorac Surg.

[3] Legras A, Guinet C, Alifano M (2012). A case of variant scimitar syndrome. Chest.

[4] Wang A, D’Amico TA, Berry MF (2014). Surgical management of congenital pulmonary malformations after the first decade of life. Ann Thorac Surg.

[5] Mikubo M, Ikeda S, Hoshino T, Yokota T, Fujii A, Mori M (2013). Pulmonary resection of lung cancer in a patient with partial anomalous pulmonary venous connection. Ann Thorac Surg.

[6] Schramel FM, Westermann CJ, Knaepen PJ, van den Bosch JM (1995). The scimitar syndrome: clinical spectrum and surgical treatment. Eur Respir J.

[7] Ramirez-Marrero MA, de Mora-Martin M (2012). Scimitar syndrome in an asymptomatic adult: fortuitous diagnosis by imaging technique. Case Rep Vasc Med.

